# MINT32: A Minimum-Image INT32 Coordinate Representation for Fast and Accurate Molecular Dynamics on GPUs

**DOI:** 10.1021/acs.jcim.5c03063

**Published:** 2026-04-04

**Authors:** Tai-Sung Lee

**Affiliations:** Laboratory for Biomolecular Simulation Research, Center for Integrative Proteomics Research, Institute for Quantitative Biomedicine (IQB), and Department of Chemistry and Chemical Biology, Rutgers University, Piscataway, New Jersey 08854, United States

## Abstract

Molecular dynamics (MD) simulations on GPUs have historically required a trade-off between performance and numerical precision. Mixed-precision approaches, such as AMBER SPFP and OpenMM, accumulate forces with high precision while representing coordinates with single precision (FP32). This design introduces a fundamental “noise floor”: quantization error in FP32 coordinates injects artificial heat into the system, degrading the long-term stability and distorting the kinetic properties. We introduce MINT32, a coordinate representation that maps the simulation box onto a 32-bit integer grid. MINT32 achieves a uniform spatial resolution of approximately 0.01 fm, roughly 2 orders of magnitude finer than the worst-case FP32 spacing. This precision comes without the memory bandwidth penalties associated with 64-bit integer arithmetic. In addition, MINT32 offers algorithmic elegance through self-wrapping, whereby periodic boundary conditions are enforced via exact integer overflow, eliminating branch divergence entirely. Benchmark results on three systems spanning 12K to 91K atoms demonstrate that MINT32 reduces energy drift in microcanonical (NVE) simulations by 5–10×, delivering double-precision-level stability for challenging PME water systems and large protein benchmarks alike. When paired with single-precision force evaluations, MINT32 outperforms conventional mixed-precision models, an observation reproduced across two independent protein systems, indicating that coordinate precision is the dominant factor governing simulation stability. The fixed-point grid further supports tightened SHAKE tolerances of 10^–7^ Å in production-length runs, with no observed convergence failures in the benchmarks reported here. Tile-matched benchmarks confirm that MINT32 integer arithmetic incurs negligible overhead (0–5%) relative to standard FP32 coordinates on consumer-grade GPUs; the 11–16% speed difference observed in the unoptimized prototype is attributable to the use of older 32 × 32 atom tiles rather than the production 16 × 16 layout. These findings serve as a conceptual foundation for future molecular dynamics (MD) engines. The primary objective of this study is to identify an optimal coordinate representation for the next-generation MD software. To this end, we present a prototype framework designed to isolate and evaluate the numerical behavior of coordinate representations in molecular dynamics, with a modified version of the AMBER simulation package serving solely as a testbed.

## Graphical abstract

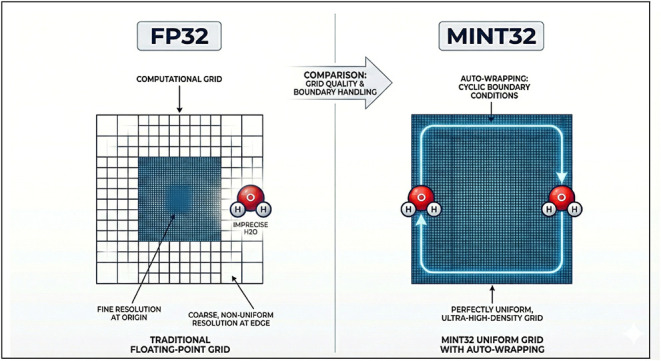

## 1 Introduction

Molecular dynamics (MD) simulations have been instrumental in the field of computational chemistry since their initial demonstration,^1^ establishing themselves as a fundamental component of modern biology by offering atomistic insights into processes such as protein folding and drug binding.^2–6^ The evolution of the field has been characterized by the pursuit of extended time scales and larger system sizes, a goal largely achieved through the integration of graphics processing units (GPUs) as the primary computational resource.^7,8^ The extensive parallelism offered by modern GPUs has facilitated routine microsecond-scale simulations, albeit with certain architectural limitations. Unlike central processing units (CPUs), which have traditionally emphasized double-precision (FP64) performance, consumer-grade GPUs are optimized for single-precision (FP32) arithmetic, often resulting in a significant reduction in FP64 throughput by factors of 32 or 64.^9–12^ Consequently, the majority of contemporary MD engines, including AMBER, GROMACS, NAMD, LAMMPS, and OpenMM, predominantly utilize FP32 arithmetic to optimize sampling efficiency.^9–11,13–15^

Nevertheless, the reliance on FP32 presents a subtle yet significant limitation: the coordinate precision. An FP32 number is defined by a 23-bit mantissa, providing approximately seven decimal digits of precision. In a typical biomolecular simulation box, such as one containing explicit water,^16^ measuring 100 Å, this results in a worst-case spatial resolution of merely a few ×10^–6^ Å near the box boundaries. Furthermore, floating-point resolution is nonuniform; the interval between representable numbers increases as particles move farther from the origin. This quantization leads to two primary sources of error. First, quantization noise in particle positions establishes a “noise floor” for the simulation state. Given that the potential energy surface is a function of coordinates, positional uncertainty directly translates to uncertainty in energy and forces, an effect exacerbated by the steep gradients of Lennard-Jones (*r* ^–12^) and Coulombic (*r* ^–1^) potentials. Second, catastrophic cancellation occurs when calculating the distance between two proximate particles with large absolute coordinates; subtracting two large, similar floating-point numbers results in a difference with markedly reduced precision.^17,18^ These artifacts manifest as noisy forces, degradation of energy conservation in microcanonical (NVE) ensembles,^19^ and instability in Particle Mesh Ewald (PME) calculations,^20–22^ where coordinate jitter introduces nondeterministic charge spreading errors.^23^ Although thermostats and barostats^24,25^ in canonical (NVT/NPT) ensembles can obscure this energy drift by dissipating the excess heat generated by numerical noise, this artificial heat sink can distort kinetics and alter the effective friction of the system, potentially biasing sensitive thermodynamic properties.

The molecular dynamics (MD) community has devised several strategies to address precision-performance trade-offs, primarily through mixed-precision floating-point models and integer-based fixed-point models. The dominant production approach, used by AMBER (SPFP), OpenMM, and GROMACS, retains FP32 for coordinate storage and pairwise distance calculations, while employing higher precision for force accumulation and integration. AMBER’s SPFP model, for example, accumulates forces in a 64-bit fixed-point format to reduce round-off error during the summation of many small terms,^9,13,26^ whereas OpenMM and GROMACS provide mixed-precision modes that integrate equations of motion in double precision or use compensated summation.^10,11^ These strategies improve accumulation accuracy, but they do not remove the more fundamental limitation that the input coordinates themselves remain quantified in FP32. High-precision forces are, therefore, still being computed from low-precision positions. Recent results in quantum chemistry reinforce the distinction between arithmetic precision and representation precision, showing that single precision is often adequate when dominant error sources are otherwise controlled.^27^

An alternative strategy is to improve the coordinate representation directly using fixed-point integers. The STORMM toolkit, for example, employs a fixed-precision INT64 coordinate representation.^28^ Mapping the simulation box to a 64-bit integer range yields subfemtometer spatial resolution, far beyond physical requirements for classical biomolecular MD, but that precision comes at a substantial cost. 64-bit integers double the bandwidth required for coordinate loads and increase register pressure on GPUs optimized for 32-bit data paths, which can reduce occupancy and overall throughput on consumer hardware. At the other end of the hardware spectrum, the Anton supercomputer uses fixed-point arithmetic for both coordinates and forces to achieve numerical stability and determinism, demonstrating that fixed-point state representations are not merely a theoretical curiosity but a practical design choice for high-performance MD architectures.^2,29,30^

The present study investigates coordinate representation as a foundational design choice for next-generation MD engines using the AMBER software package strictly as a convenient testbed. The implementation is intentionally limited to the minimum functionality required to test our hypotheses because our objective is to isolate and quantify the effects of coordinate precision on simulation stability rather than to improve the existing AMBER engine. The resulting AMBER implementation is, therefore, a minimal research prototype designed to generate controlled data for evaluating the MINT32 concept. Production-quality integration of MINT32-style representations into a modern MD engine would require substantial additional engineering beyond the scope of the present work.

In this study, we introduce **MINT32**, a coordinate representation that occupies an optimal position between the inadequate resolution of FP32 and the high computational cost of INT64. MINT32 maps the periodic simulation box to the entire range of a 32-bit signed integer using the fixed scaling factor *S* = 2^32^/*L*. By foregoing the unnecessary dynamic range of floating-point numbers, which can represent both galaxies and atoms, in favor of a fixed-point grid specifically tailored to the simulation box, MINT32 achieves a uniform spatial resolution of approximately 2 × 10^–8^ Å for a 100 Å system. Depending on box size and coordinate origin, this represents an improvement of roughly 2 orders of magnitude over the worst-case FP32 spacing, sufficient to suppress coordinate noise below the level of other integration errors. Additionally, this scheme naturally exploits the inherent periodic wrapping of integer arithmetic (“self-wrapping”) to manage minimum-image conventions,^31^ thereby simplifying the logic in force kernels and eliminating branch divergence. We present the theoretical foundation of MINT32, its implementation within the AMBER software package, and benchmarks on three systems (4096 water, DHFR, and Factor IX) demonstrating that it minimizes PME grid noise and supports tightened SHAKE^32^ and RATTLE^33^ constraint tolerances of 10^–7^ Å in production-length runs. In microcanonical benchmarks, MINT32 reduces energy drift by 5–10× compared to standard production settings across all tested systems, highlighting that coordinate precision, not force accumulation, is the primary bottleneck for long-time scale GPU simulations.^34^

The benchmarks reported here are therefore intended to be illustrative rather than exhaustive. Because AMBER is a mature and tightly integrated codebase, introducing a new coordinate representation into its critical infrastructure necessarily entails both technical compromises and implementation risk. Performance optimization, broader validation beyond the example systems studied here, and extension to production-style applications are left for future work.

## 2 Theory

### 2.1 Coordinate Representation and Precision

The primary challenge in representing atomic coordinates for molecular dynamics lies in achieving a balance between the range and resolution. In the standard IEEE-754 single precision (FP32) format, a coordinate *x* is expressed as

1 $$x={\left(-1\right)}^{s}\times 1.m\times 2^{e-127}$$

where *s* denotes the sign bit, *m* represents the 23-bit mantissa, and *e* is the 8-bit exponent. This format is engineered to accommodate an extensive dynamic range (∼10^38^), enabling the representation of values from the subatomic to galactic scales. However, for a typical molecular dynamics (MD) simulation box, which seldom exceeds a few hundred Ångstroms, this expansive range is largely underutilized.

FP32 provides roughly 24 bits of significant precision, so its absolute coordinate spacing depends on the coordinate magnitude. For coordinates of order 1 Å, the spacing is about 2^–23^ to 2^–24^ Å, whereas near the edge of a 100 Å box, it is about 2^–17^ Å. Thus, displacements formed by subtracting large coordinates can suffer from both coarse absolute spacing and cancellation. This nonuniformity implies that the physics of the simulation is contingent upon the particle’s location within the box, thereby violating translational invariance.

Moreover, computing the distance Δ*x* = *x*_*i*_ – *x*_*j*_ between two proximate particles with large absolute coordinates can result in catastrophic cancellation. Consider two particles positioned at *x*_*i*_ = 50.00000 and *x*_*j*_ = 50.00001. In FP32, these values may be rounded to the same representable number, yielding a calculated distance of zero, or to adjacent representable numbers, resulting in a discrete step. This loss of significant bits severely diminishes the precision of the resulting difference, introducing noise precisely where accuracy is paramount: in the calculation of short-range interactions. This FP32-centric configuration underpins the default GPU paths in widely used engines such as GROMACS and OpenMM.^10,11^

#### 2.1.1 MINT32 Representation

The MINT32 framework addresses the aforementioned challenges by employing a fixed-point integer representation with a specified scale. For an orthorhombic box characterized by side lengths *L*_*x*_, *L*_*y*_, *L*_*z*_, each dimension is mapped to the complete signed 32-bit range as follows

2 $$X_{\alpha }=\mathrm{round}\left(x_{\alpha }\cdot S_{\alpha }\right),,S_{\alpha }=\frac{2^{32}}{L_{\alpha }}$$

where α ∈ {*x*, *y*, *z*}. Within this framework, the spatial resolution is exclusively determined by *S*_α_ and remains consistent throughout the simulation box:

3 $$\delta x_{\alpha ,\mathrm{MINT}}=\frac{1}{S_{\alpha }}=\frac{L_{\alpha }}{2^{32}}$$

This consistency reinstates translational invariance with the coordinate grid.

The selection of a 32-bit integer represents an optimal compromise. A 16-bit integer would provide inadequate resolution (approximately 0.001 Å for a 100 Å box), whereas a 64-bit integer, despite offering subfemtometer precision, would incur substantial performance penalties on consumer GPUs, where 64-bit integer operations share the severely limited FP64 execution units (typically 1:32 or 1:64 throughput relative to 32-bit operations) and also double register pressure and memory bandwidth requirements. In contrast, MINT32 maps the simulation box onto a uniform 32-bit integer lattice, giving a box-wide spacing of *L*/2^32^. For *L* = 100 Å, this is about 100/2^32^ ≈ 2.3 × 10^–8^ Å everywhere in the box. At the box edge, this represents an improvement of roughly 2 orders of magnitude over the FP32 spacing; the exact ratio depends on box size and coordinate origin (Table 1). For a centered 100 Å box, the worst-case FP32 spacing at coordinate ±50 Å is 2^–18^ ≈ 3.8 × 10^–6^ Å, yielding a ratio of approximately 160 ×; for an uncentered box, the ratio can exceed 300×. Table 1 provides a summary of this comparison across typical system sizes.

**Table 1:** Spatial Resolution Comparison between FP32 and MINT32 for Various Simulation Box Sizes^a^

	FP32 worst case	MINT32 uniform	improvement	
box size (*L*) (Å)	(at *x* = *L*) (Å)	(*L*/2^32^) (Å)	uncentered	centered
30	1.9 × 10^–6^	7.0 × 10^–9^	∼270×	∼135×
100	7.6 × 10^–6^	2.3 × 10^–8^	∼330×	∼160×
300	3.1 × 10^–5^	7.0 × 10^–8^	∼440×	∼220×

a FP32 worst-case spacing is evaluated at the box edge for an uncentered [0, *L*] box; for a centered [−*L*/2, *L*/2] box, the worst-case coordinate is *L*/2 and the ratio is correspondingly smaller.

#### 2.1.2 Box Geometry and Scaling

For an orthorhombic box with side lengths *L*_*x*_ ≠ *L*_*y*_ ≠ *L*_*z*_, each axis uses its own independent scale factor *S*_α_ = 2^32^/*L*_α_. The absolute spatial resolution, therefore, differs between axes, as a longer axis has coarser resolution, but the resolution is strictly uniform within each axis and does not vary with particle position. This position-independence is the fundamental advantage over FP32, where resolution degrades with increasing coordinate magnitude regardless of axis. The fixed per-axis scaling *S*_α_ = 2^32^/*L*_α_ is not an arbitrary tuning choice: it is the specific mapping that makes one periodic box length coincide with the full 2^32^ integer cycle, thereby preserving the self-wrapping behavior described below while eliminating any adjustable degree of freedom.

#### 2.1.3 Extension to Nonorthorhombic Cells

For a general periodic cell defined by the cell matrix *H* (whose columns are the lattice vectors **a**_1_, **a**_2_, and **a**_3_), the MINT32 representation operates on fractional coordinates **u** = *H* ^–1^ **r**, with each component mapped to the full signed 32-bit range

4 $$U_{\alpha }=\mathrm{round}\left(u_{\alpha }\cdot 2^{32}\right),,u_{\alpha }\in \left[-\frac{1}{2},\frac{1}{2}\right)$$

The minimum-image fractional displacement is computed by exact integer subtraction Δ*U*_α_ = *U*_*i*,α_ – *U*_*k*,α_, with two-complement overflow providing automatic periodic wrapping along each lattice direction, exactly as in the orthorhombic case. The Cartesian displacement is then recovered as Δ**r** = *H*·Δ**u**, where Δ*u*_α_ = float­(Δ*U*_α_)/2^32^.

We illustrate the feasibility and precision of this approach using the truncated octahedron, the most common nonorthorhombic cell in biomolecular simulation. For the standard AMBER truncated octahedron (α = β = γ = arccos­(−1/3) ≈ 109.47°), the cell matrix takes the upper-triangular form (eq 5)­

5 $$H=a\left(\begin{array}{lll} 1 & -\frac{1}{3} & -\frac{1}{3}\\ 0 & \frac{2\sqrt{2}}{3} & -\frac{\sqrt{2}}{3}\\ 0 & 0 & \frac{\sqrt{6}}{3} \end{array}\right)$$

where *a* is the box length parameter. Using standard IEEE-754 error analysis, the total reconstruction error per Cartesian component is bounded by (eq 6)­

6 $$|\mathrm{err}\left(\Delta r_{i}\right)|\leq \gamma_{2}\sum_{j}\left|H_{\mathrm{ij}}\right|\left|\Delta u_{j}\right|+\sum_{j}\left|H_{\mathrm{ij}}\right|\left(\frac{\eta_{j}}{2^{32}}+\left|\Delta u_{j}\right|\cdot u\right)$$

where *u* = 2^–24^ is the FP32 unit roundoff, γ_2_ ≈ 2*u*, and η_*j*_ is the INT32→FP32 rounding error. For a DHFR-sized truncated octahedron (*a* = 56.5 Å) at the 8 Å nonbonded cutoff, this yields a worst-case displacement error of ∼4 × 10^–6^ Å per component, comparable to the FP32 coordinate spacing at the box edge and confirmed by Monte Carlo sampling. The dominant error source is the same INT32→FP32 conversion “staircase effect” present in the orthorhombic case; the cell-matrix multiplication does not significantly amplify it (κ­(*H*) = 2). Our prototype is designed for orthorhombic cells; a full triclinic implementation, including PME grid mapping and constraint geometry modifications, is left for future work.

#### 2.1.4 Rounding and Quantization Noise

Coordinates are rounded to the nearest integer, employing a tie-to-even approach in hardware, which results in a quantization error uniformly distributed within the interval [−0.5, 0.5]/*S*_α_, with a variance of σ^2^ = (12 *S*_α_^2^)^−1^. The rounding process is unbiased, and as a result, the noise floor is deterministically reduced by increasing *S*_α_ (i.e., by decreasing *L*_α_ or operating at the 32-bit dynamic limit). During barostat moves (NPT ensemble), the box scaling is handled by updating *S*_α_; the integer coordinates *X*_α_ remain invariant (consistent with *x*_α_/*L*_α_ = const), thereby preventing the accumulation of rounding errors associated with coordinate rescaling.

### 2.2 Minimum-Image Convention

A significant feature of MINT32 is its proficient management of the periodic boundary conditions (PBC). In conventional floating-point molecular dynamics (MD), determining the minimum-image separation Δ*x* necessitates verifying whether the raw difference surpasses half the box length, followed by applying a correction. This process typically involves conditional logic (branching) or rounding operations

dx=x[i]−x[j];dx−=round(dx/L)×L

Such operations can be computationally expensive in a GPU kernel, where thread divergence (branching) adversely affects performance. In MINT32, this simplification depends directly on using the full-range mapping *S*_α_ = 2^32^/*L*_α_; if one instead chose an arbitrary fixed scale unrelated to the box length, explicit wrapping logic would be required again, and the central self-wrapping advantage would be lost.

#### 2.2.1 Automatic Periodic Boundary Handling

In the MINT32 framework, each box length *L*_α_ is precisely mapped to the entire span of the 32-bit integer range (2^32^). Consequently, the periodic boundary aligns exactly with the integer overflow limit. The minimum image difference is calculated component-wise as

7 $$\Delta X_{\alpha }=X_{i,\alpha }-X_{j,\alpha }$$

utilizing standard 32-bit integer subtraction. Due to the characteristics of two’s complement arithmetic, if the distance between two particles exceeds half the box size along any axis (i.e., crosses the periodic boundary), the result naturally overflows and wraps around into the correct signed range [−2^31^, 2^31^ −1]. This method yields the correct minimum-image displacement automatically, with no branching or correction logic required.

This design obviates the necessity for explicit wrapping code (e.g., “if” statements or modulus operations) in the distance calculation, thereby streamlining the inner loops of the force kernels. The subtraction Δ*X* = *X*_*i*_ – *X*_*j*_ is exact and immune to the cancellation errors that afflict FP32. Since addition and subtraction operations on coordinates are automatically wrapped, the positions of all atoms will consistently be autowrapped.

#### 2.2.2 Concrete Example

Consider two particles within a box of dimensions *L*_*x*_ = 50 Å, positioned at *x*_*i*_ = −24.9 Å (proximal to the left boundary) and *x*_*j*_ = +24.9 Å (proximal to the right boundary). The straightforward calculation of the difference yields *x*_*j*_ – *x*_*i*_ = 49.8 Å, which approximates the entire length of the box—an evidently incorrect result for the minimum image, which should be 0.2 Å across the periodic boundary.

In the MINT32 framework, the scale factor is defined as *S* = 2^32^/50 ≈85,899,346. The integer coordinates are computed as follows

8 $$X_{i}=\mathrm{round}\left(-24.9\times S\right)=-2138926693$$

9 $$X_{j}=\mathrm{round}\left(+24.9\times S\right)=+2138926693$$

The direct integer subtraction results in *X*_*j*_ – *X*_*i*_ = 4,277,853,386, which surpasses 2^31^ = 2,147,483,648, thereby causing an overflow in the signed 32-bit range. Due to two’s complement wraparound, the outcome is −17,113,910, which corresponds to

10 $$\Delta x=\frac{-17113910}{85899346}\approx -0.2\;Å$$

This represents the accurate minimum-image displacement achieved automatically without the need for conditional logic.

The final floating-point displacement utilized for force calculation is derived through a single multiplication per component

11 $$\Delta x_{\alpha ,\mathrm{float}}=\mathrm{float}\left(\Delta X_{\alpha }\right)\cdot S_{\alpha }^{-1}$$

This methodology ensures preservation of the high-precision integer difference until the final stage of computation.

### 2.3 Numerical Stability in Force Calculation

The enhanced resolution of coordinates significantly influences the stability of the force calculations, especially concerning short-range interactions. The force error δ*F* arising from a coordinate error δ*r* can be approximated by the derivative of the potential

12 $$\delta F\approx \left|\frac{dF}{dr}\right|\delta r=\left|\frac{d^{2}V}{dr^{2}}\right|\delta r$$

For the Lennard-Jones potential, *V*_LJ_ ∝ *r* ^–12^, the force scales as *F* ∝ *r* ^–13^, and the stiffness (second derivative) scales as *r* ^–14^. At short interatomic distances, this stiffness term becomes exceedingly large. Although the FP32 coordinate spacing near the box edge is on the order of 10^–6^–10^–5^ Å (depending on box size), the effective displacement error after catastrophic cancellation between nearby particles with large absolute coordinates can be substantially larger. By providing a uniform spacing of approximately 1 × 10^–8^ Å, MINT32 suppresses this noise at its source, reducing coordinate-derived force errors by several orders of magnitude.

#### 2.3.1 Precision Loss in Bonded Terms

The impact of coordinate precision is particularly significant in bonded interactions and geometric constraints. Consider the harmonic bond potential *V* = *k*(*r* – *r*_0_)^2^, where the force calculation depends on the difference between the current bond length *r* and the equilibrium length *r*_0_. For atoms located far from the origin, such as those near the periphery of a large simulation box, the absolute coordinates are substantial. In FP32, these coordinates experience a loss of 2–3 significant digits of precision. This loss propagates when calculating the bond length $r=\sqrt{{\left(x_{i}-x_{j}\right)}^{2}+\cdot \cdot \cdot }$ . Critically, when computing the deviation (*r* – *r*_0_), the subtraction of two nearly identical numbers (e.g., *r* ≈ 1.000002 Å and *r*_0_ = 1.000000 Å) is involved. If *r* has already suffered precision loss due to the large absolute coordinates of the atoms, then the resultant difference is predominantly noise. For instance, calculating 1.000001222–1.000002232 necessitates high precision in the inputs. If the inputs are truncated by FP32 quantization at the box edge, the resulting force is essentially random. This renders standard FP32 coordinates inadequate for high-fidelity bonded interactions in large systems.

Conversely, the MINT32 grid significantly reduces coordinate uncertainty by approximately 4 orders of magnitude. For a 100 Å box, the per-component standard deviation of the rounding error is approximately 10^–8^ Å, consistent with the tightened 10^–7^ Å SHAKE tolerance used in the benchmarks presented here and with reduced catastrophic cancellation relative to FP32.

Additionally, a “staircase effect” was identified when converting integer differences to a floating point. If the integer difference Δ*X* is directly converted to a single-precision float before scaling (float­(Δ*X*)·*S* ^–1^), the limited significand of the float can reintroduce quantization steps for large separations. Theoretically, the optimal solution is to promote the integer difference to double the precision before scaling:

13 $$\Delta x_{\mathrm{float}}=\mathrm{float}\left(\mathrm{double}\left(\Delta X\right)\cdot S^{-1}\right)$$

This approach preserves an additional 3–5 bits of precision compared to the direct float conversion. However, as discussed in the Implementation section, the cost of double-precision multiplication in the inner force loop is prohibitive on consumer GPUs. Consequently, our implementation employs the direct floating conversion path. Despite this theoretical precision loss, our results indicate that the MINT32 representation still offers a substantial stability improvement over standard FP32 coordinates, effectively mitigating the dominant noise sources.

### 2.4 Representation vs Accumulation

It is essential to differentiate between coordinate representation (the method of storing positions) and force accumulation (the process of summing forces). MINT32 serves solely as a representation strategy and is independent of the accumulation method.

- **AMBER SPFP:** Utilizes FP32 coordinates (limited precision) with fixed-point accumulation.
- **STORMM:** Employs INT64 coordinates (high precision) and INT64 accumulation.
- **MINT32:** Implements INT32 coordinates (high precision) and is compatible with FP32, FP64, or fixed-point accumulation.

By standardizing the input precision, MINT32 ensures that even conventional FP64 accumulation receives precise inputs, thereby mitigating the “garbage in, high-precision garbage out” issue.

### 2.5 Impact on PME Stability

In the Particle Mesh Ewald (PME) method, particles are mapped to a grid using B-spline interpolation, which depends on the fractional coordinates *u* = *x*/*L*. In an FP32 representation, quantization noise in *x* directly translates to jitter in *u*. Given that charge assignment is highly sensitive to the precise position within a grid cell, this jitter results in a nondeterministic charge distribution and noise in the reciprocal space energy. MINT32 offers stable, high-resolution fractional coordinates, ensuring that the PME charge mapping remains robust and deterministic.

The fractional jitter under MINT32 is constrained by $\sigma_{u}\approx {\left(\sqrt{12}2^{32}\right)}^{-1}$ per component (i.e., $O\left(10^{-10}\right)$ of the box length), ensuring that the spline weights and their phase factors are effectively deterministic even for large systems. This reduces the noise floor in the reciprocal space and accounts for the significant reduction in drift observed in homogeneous water boxes. A quantitative analysis of the particle-to-mesh alignment error (Section 4.3) confirms that MINT32 reduces the worst-case fractional grid-position uncertainty by a factor of 130–230× relative to FP32 for the systems studied, suppressing the per-particle B-spline weight error from order 10^–7^–10^–6^ to order 10^–9^–10^–8^.

## 3 Implementation

We integrated the MINT32 coordinate representation into the pmemd.cuda engine of the AMBER molecular dynamics package. This integration is deliberately minimal and unoptimized, serving solely as a proof-of-concept to validate the coordinate representation. Retrofitting a fundamental data type into a mature, highly optimized codebase presents significant challenges. The AMBER GPU engine relies on intricate memory access patterns and optimized 16 × 16 atom tile layouts for 32-bit floating-point data. Our implementation strategy focused on minimally invasive modifications that isolate the high-precision integer logic within the core force and integration kernels, while maintaining compatibility with the surrounding infrastructure.

### 3.1 Data Structures and Memory Layout

The standard AMBER SPFP engine organizes atomic coordinates in global memory using Structure-of-Arrays (SoA) layouts, typically employing separate float arrays for *x*, *y*, *z*, or interleaved float3 arrays. To accommodate MINT32, we introduced a new global array, int4* pAtomCoord, which serves as the definitive source for particle positions.

#### 3.1.1 int4 Vector Type

The selection of the CUDA int4 vector type reflects several practical considerations. Its 128-bit alignment enables fully coalesced global memory access, thereby maximizing bandwidth utilization on NVIDIA GPUs. The first three components (*x*, *y*, *z*) store the fixed-point coordinates, while the fourth component (*w*) is presently reserved as padding but also provides a zero-cost location for static atomic properties such as atom-type or charge indices that would otherwise require separate fetches. In addition, loading one 128-bit vector is typically more efficient than issuing three separate 32-bit loads, reducing the number of memory transactions required per warp.

#### 3.1.2 Dual-State Synchronization

To ensure compatibility with the extensive range of AMBER features, including restraints, analysis hooks, and complex barostats, we adopted a “dual-state” strategy. The primary state is the pAtomCoord array, which contains high-precision integer coordinates and serves as the reference representation for force calculations and time integration. The legacy floating-point arrays are retained as a secondary state, effectively functioning as a cache that is refreshed from the MINT32 representation only when needed by routines not yet ported to integer arithmetic. This decoupling allows kernels to be migrated incrementally to MINT32 without destabilizing the surrounding engine infrastructure.

### 3.2 Coordinate Representation across the Time step

Figure 1 provides a schematic view of how coordinates move through a single time step in the baseline pmemd.cuda SPFP engine and the MINT32 prototype. Table 2 then summarizes the corresponding representation choices for the baseline AMBER engines, the MINT32 prototype, and the literature-described comparison engines. The key distinction is that the MINT32 prototype uses INT32 as the authoritative state and performs all coordinate differences via exact integer subtraction, whereas the baseline engines store coordinates in FP64 and derive FP32 or FP64 working copies for each kernel.

**Figure 1:**
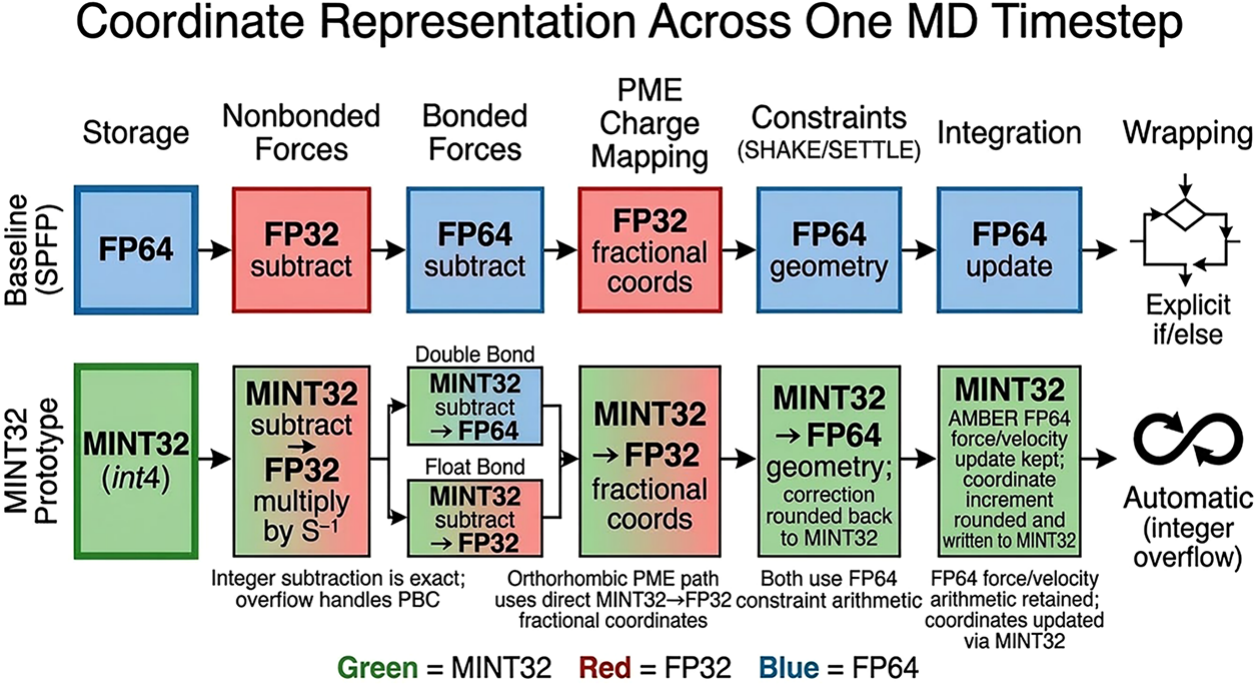
Coordinate representation across one MD time step for the baseline AMBER SPFP engine (top) and the MINT32 prototype (bottom). Colors indicate precision: green = INT32, red = FP32, and blue = FP64. In the MINT32 workflow, INT32 is the authoritative coordinate state; nonbonded differences are formed by exact integer subtraction, bonded terms are shown in both Double Bond ("INT32 → FP64") and Float Bond ("INT32 → FP32") variants, orthorhombic PME charge mapping derives FP32 fractional coordinates directly from MINT32, and integration retains FP64 force/velocity arithmetic while writing updated coordinates back through MINT32. Periodic boundary wrapping is handled automatically via an integer overflow.

**Table 2:** Coordinate Representation at Each Stage of the MD Time step^a^

stage	SPFP	DPFP	MINT32	OpenMM^b^	STORMM^c^
storage	FP64	FP64	MINT32	FP64 or FP32	INT64+INT32
NB displ.	FP32	FP64	MINT32→FP32	FP32	INT95
bonded displ.	FP32	FP64	MINT32→FP64	FP32	INT95
PME frac. coords	FP32	FP64	MINT32→FP32^d^	FP32	INT95
constraints	FP64	FP64	MINT32→FP64	FP32 or FP64	not publ.
integration	FP64	FP64	FP64→MINT32	FP64	INT95
wrapping	explicit	explicit	overflow	explicit	not publ.

a AMBER SPFP, DPFP, and the MINT32 prototype are described from the implementation; OpenMM and STORMM entries are from published descriptions.^11,28^

b OpenMM 8.x mixed-precision mode.^11^ Constraint precision depends on the user-selected platform and precision mode.

c STORMM uses a 95-bit fixed-precision format (INT64+INT32) for coordinates and arithmetic; wrapping model details are not described in the published ref 28.

d Current orthorhombic non-NPT MINT32 PME maps centered MINT32 coordinates directly to FP32 fractional coordinates; the legacy FP64 mirror path is retained as a fallback for NPT and nonorthogonal boxes.

### 3.3 Modifications to Nonbonded Kernels

The nonbonded force calculation, exemplified by kNLCPNE.h, represents the performance-critical component in AMBER, typically dominating the total runtime. In the standard implementation, threads load neighbor coordinates as floats. In MINT32, we altered the kernel to load int4 vectors directly.

#### 3.3.1 Precision-Preserving Distance Calculation

The calculation of the distance between atom *i* and atom *j* is conducted entirely within integer space

14 $$\Delta X_{\mathrm{ij}}=X_{i}-X_{j}$$

As elaborated in the Theory section, this integer subtraction is exact and resistant to catastrophic cancellation. However, converting this difference to a floating-point distance for force evaluation necessitates careful consideration.

As discussed in the Theory section, directly converting the integer displacement to FP32 can result in a “staircase effect” when the mantissa is unable to resolve the fine integer grid for large separations. The numerically optimal approach involves promoting the integer difference to FP64 before scaling and downcasting to FP32 solely for the force lookup

15 $$\Delta x_{\mathrm{float}}=\mathrm{float}\left(\mathrm{double}\left(\Delta X_{\mathrm{ij}}\right)\times S_{\mathrm{double}}^{-1}\right)$$

In practice, double-precision multiplication within this inner kernel proved too costly on consumer GPUs. Consequently, we employ the streamlined approach

16 $$\Delta x_{\mathrm{float}}=\mathrm{float}\left(\Delta X_{\mathrm{ij}}\right)\times S_{\mathrm{float}}^{-1}$$

which retains the core advantage of the integer representation. The selection of this simpler single-precision conversion path reflects the scope of this work as a proof-of-concept; no effort was made to optimize this path for performance or precision beyond what was necessary for validation. As shown in the Results section (Section 4.5), tile-matched benchmarks confirm that neither the integer subtraction nor this FP32 conversion introduces measurable overhead relative to the standard SPFP kernel. Benchmarks indicate that any residual staircase artifacts are negligible compared to the original FP32 quantization noise; internal testing confirmed that promoting to double precision did not yield a statistically significant improvement in energy drift while incurring a substantial performance penalty.

#### 3.3.2 Automatic Periodic Boundary Handling

A significant architectural advantage of the MINT32 representation is its inherent “self-wrapping” capability. In conventional floating-point molecular dynamics (MD), the enforcement of periodic boundary conditions (PBC) necessitates explicit conditional checks or computationally expensive modulus operations at each step to ensure that particles remain within the primary unit cell. With MINT32, the simulation box is mapped to the entire range of the 32-bit signed integer (−2^31^ to 2^31^–1). Consequently, the natural overflow behavior of two’s complement arithmetic automatically manages PBC wrapping. When a particle crosses a boundary, its integer coordinate naturally wraps around to the opposite side of the range, precisely corresponding to entering the opposite side of the simulation box.

This feature enabled us to significantly simplify the AMBER codebase. The molecular system is wrapped into the primary cell only once, at initialization, after which all explicit wrapping logic can be removed from the integration and force kernels. The MINT32-modified kernels therefore contain no “if” statements or “fmod”; calls for boundary enforcement, and the wrapping operation itself is handled directly by the arithmetic unit at effectively zero computational cost, eliminating a common source of warp divergence.

### 3.4 Constraints (SHAKE and RATTLE)

Geometric constraints, such as the fixation of hydrogen bond lengths, exhibit a particular sensitivity to coordinate precision. The conventional SHAKE algorithm iterates until the bond constraint *r* ^2^ – *d*_0_^2^ ≈ 0 is satisfied. In FP32, atoms located near the periphery of the simulation box experience diminished spatial resolution, resulting in the loss of 2–3 significant digits. This error is exacerbated when calculating the current bond length squared (*r* ^2^). The convergence check involves comparing this imprecise value of *r* ^2^ against the target *d*_0_^2^. Given the proximity of these values, subtraction *r* ^2^ – *d*_0_^2^ is prone to catastrophic cancellation. For instance, differentiating a bond length of 1.000001 Å from 1.000002 Å is unfeasible if the coordinates themselves possess an uncertainty of ±0.00001 Å. Consequently, FP32 SHAKE implementations are frequently constrained to a tolerance of approximately 10^–5^ Å.

We restructured the SHAKE kernel to function directly on the MINT32 arrays. We enhanced the simulation state with a pOldAtomCoord array to retain the integer positions from the preceding time step. The integer-based SHAKE algorithm proceeds as follows:

1 **Load:** Retrieve current (*X*(*t*)) and previous (*X*(*t* – δ*t*)) positions as int4 from pAtomCoord_M32 and pOldAtomCoord_M32.
2 **Compute Unconstrained Vector:** Determine the bond vector through integer subtraction, yielding the exact minimum-image displacement devoid of PBC noise. The integer difference is promoted to double precision before scaling: Δ*x* = (double)­Δ*X* · *S* ^–1^.
3 **Iterate:** All constraint geometry (reference vectors, current distances, and correction magnitudes) is computed in FP64. The correction displacement is converted back to the integer domain via Δ*X*_corr_ = (int)­rint­(Δ*x*_corr_ · *S*) and applied directly to the integer coordinates.

SETTLE (for rigid water molecules) follows the same pattern: integer coordinates provide the input vectors, all internal rotational geometry is computed in FP64 (as in the baseline pmemd.cuda SETTLE), and the corrected displacements are rounded back to INT32. RATTLE velocity constraints also use FP64 arithmetic, with INT32 coordinates used only for the distance differences.

By anchoring the constraint solver to the integer grid and performing internal arithmetic in FP64, the prototype sustains SHAKE tolerances of 10^–7^ Å in production-length benchmark runs, which is 2 orders of magnitude more stringent than the standard FP32 implementation.

### 3.5 Integration and Barostats

The integration kernel (kU.h) is tasked with advancing both the positions and velocities. Within MINT32, this kernel undertakes the essential function of converting velocities, which are maintained in FP32, into integer displacements.

17 X(t+δt)=X(t)+round(v(t+δt/2)·δt·S)

It is crucial to recognize that scaling factor *S* is not an arbitrary parameter. It is precisely defined as *S* = 2^32^/*L*, thereby mapping the physical box dimension *L* to the complete 32-bit integer range. This definition ensures that the integer overflow logic appropriately manages periodic boundaries.

The management of constant pressure (NPT) ensembles introduces an additional layer of complexity: box scaling. In principle, when the box volume changes, only the scale factor needs updating

18 $$S_{\mathrm{new}}=\frac{2^{32}}{L_{\mathrm{new}}},,X_{\mathrm{new}}=X_{\mathrm{old}}$$

effectively rescaling the physical positions *x* = *X*/*S* by reinterpreting the integer grid without modifying the integer values themselves. This design avoids the accumulation of rounding errors during volume changes and preserves the exact minimum-image property. In the current prototype, this direct path is not yet implemented; instead, the legacy barostat code rescales the FP64 Cartesian coordinates (pImageX/Y/Z), which are then reconverted to MINT32 using the new scale factor. This introduces one additional quantization step per barostat move but preserves compatibility with the existing AMBER barostat infrastructure. All benchmarks in this study use NVE ensembles and do not exercise either path.

## 4 Results

We conducted an evaluation of the MINT32 implementation utilizing three benchmark systems: a pure solvent box comprising 4096 TIP3P water molecules, a solvated protein (dihydrofolate reductase, DHFR), and a larger solvated protein (coagulation Factor IX). All simulations were executed within the microcanonical electron microscopy (NVE) ensemble. The NVE ensemble is considered the benchmark for assessing numerical precision, as, in the absence of a thermostat, the total energy of the system should remain invariant. Any deviation in total energy serves as a direct indicator of numerical noise, specifically the nonconservative work resulting from quantization errors in forces and positions. These tests directly examine the hypothesis posited in the Theory section, which suggests that fixed-grid coordinates rather than force accumulation precision establish the predominant noise floor on GPUs.

The three systems were chosen to probe complementary aspects of coordinate quantization. The pure water box, with its high density of diffusible dipoles, isolates the sensitivity of the particle mesh Ewald (PME) grid and the accuracy of minimum-image wrapping across periodic boundaries. The DHFR system exemplifies a standard biomolecular simulation characterized by heterogeneous forces and tests the stability of rigid bond constraints (SHAKE) and bonded interactions under conditions of limited precision. The Factor IX system provides a substantially larger protein system (∼4 × the atom count of DHFR) with a different force field composition, testing whether the DHFR observations, particularly the counterintuitive float-bond advantage, generalize beyond a single protein. These benchmarks are intended to illustrate numerical behavior rather than provide a comprehensive performance characterization of the AMBER engine.

### 4.1 Computational Methods

#### 4.1.1 Water System

A cubic box containing 4096 TIP3P water molecules (12,288 atoms) serves as a stress test for nonbonded interactions, given the high density of mobile dipoles, which renders the energy particularly sensitive to PME accuracy. **DHFR System:** The JAC benchmark system (Joint AMBER-CHARMM) comprises DHFR in explicit water, consisting of 22,930 atoms within a 62 × 62 × 62 Å^3^ box. This system exemplifies a typical biological simulation, characterized by a heterogeneous mixture of stiff bonded terms (protein backbone) and a bulk solvent. Hydrogen Mass Repartitioning (HMR) was employed to facilitate a 4 fs time step in standard production; however, a reduced time step of 1 fs was utilized for NVE stability testing to rigorously isolate coordinate noise from integration errors. **Baseline Context:** This DHFR benchmark has been previously employed to compare the AMBER GPU DPFP and SPFP paths;^35^ it is reused here to isolate the impact of coordinate representation. **Factor IX System:** Coagulation Factor IX solvated in explicit water, comprising 90,906 atoms (392 protein residues including γ-carboxyglutamic acid modifications, 28,358 TIP3P water molecules) within a 142 × 83 × 79 Å^3^ orthorhombic box. This system is approximately four times larger than DHFR and features a different protein architecture and force field composition, providing an independent test of the float-bond observation. **Input Files:** For the water and DHFR systems, the input files located in the test/cuda/directories of the AMBER suite were directly utilized. **Hardware:** All SPFP and MINT32 benchmarks were executed on a single NVIDIA RTX 3090 GPU. DPFP benchmarks were conducted on a single NVIDIA Titan V GPU, whose 1:2 FP64:FP32 throughput ratio makes it particularly suitable for double-precision calculations (consumer GPUs such as RTX 3090 have a 1:64 ratio, rendering DPFP benchmarks impractically slow). Both GPUs utilized the CUDA version 12.9 toolkit. The reference “Master” simulations employed the 11/09/2025 snapshot of the master branch of the AMBER codebase repository (FP32 coordinates, double-precision bonded terms, and fixed-point accumulation).

#### 4.1.2 Simulation Protocol

All benchmarks adhered to a standard microcanonical (NVE) protocol to rigorously conserve energy, employing a time step of 1.0 fs and a nonbonded interaction cutoff of 8.0 Å. Constraints on bonds involving hydrogen were enforced using SHAKE (for the DHFR system) and SETTLE (for rigid TIP3P water molecules in all three systems) with a tightened SHAKE tolerance of 10^–7^ Å (in contrast to the default of 10^–5^ Å) to minimize constraint-related noise. The PME grid dimensions were explicitly defined to ensure consistency across runs, and the net force removal flag was disabled (netfrc = 0). The 4096 Water system was simulated for 100 ns (1 × 10^8^ steps) to provide robust statistics on long-term drift, while the DHFR and Factor IX systems were each simulated for 50 ns (5 × 10^7^ steps).

### 4.2 Energy Conservation

The principal criterion for assessing coordinate precision is the long-term stability of the total energy. In an ideal NVE simulation, the total energy should oscillate around a mean value due to symplectic integration errors without exhibiting a systematic drift. However, quantization noise in particle positions introduces a stochastic, nonconservative force that gradually injects or removes energy from the system. Table 3 presents a summary of the drift rates and performance for all of the systems. Drift values are reported in units of *k*_B_ *T*/ns per degree of freedom (DOF) at 300 K, facilitating a normalized comparison across systems of varying sizes.

**Table 3:** Energy Drift and Performance Comparison^a^

system	implementation	drift (*k*_B_ *T*/ns/DOF)	stability	speed (ns/day)
water	Master (DPFP)	2.09 × 10^–5^	1.00	175.9
water	Master (SPFP)	2.22 × 10^–4^	0.09	495.2 (100%)
water	MINT32	2.22 × 10^–5^	0.94	415.0 (84%)
DHFR	Master (DPFP)	–8.74 × 10^–6^	1.00	116.6
DHFR	Master (SPFP)	–4.64 × 10^–5^	0.19	288.3 (100%)
DHFR	MINT32 (bond = double)	–1.46 × 10^–5^	0.60	250.2 (87%)
DHFR	MINT32 (bond = float)	–9.25 × 10^–6^	0.94	255.5 (89%)
Factor IX	Master (DPFP)	3.78 × 10^–6^	1.00	38.2
Factor IX	Master (SPFP)	3.48 × 10^–5^	0.11	121.5 (100%)
Factor IX	MINT32 (bond = double)	6.43 × 10^–6^	0.59	106.1 (87%)
Factor IX	MINT32 (bond = float)	4.24 × 10^–6^	0.89	108.3 (89%)

a Drift is reported in *k*_B_ *T*/ns per Degree of Freedom (DOF) at 300 K.

#### 4.2.1 Pure Solvent: The PME Stress Test

In the case of the pure water system (Figure 2), the enhancement was even more significant. Water boxes are notoriously challenging for energy conservation due to the rapid rotation of water molecules and the high density of partial charges, which impose a substantial burden on the PME solver.

**Figure 2:**
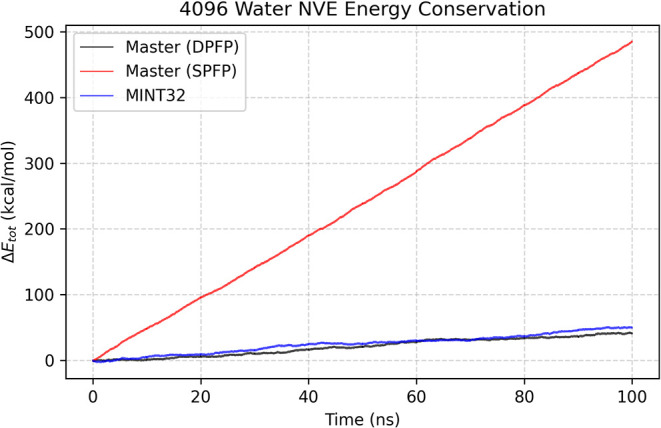
Total energy conservation for the 4096-well water system (NVE). MINT32 (blue) matches the stability of the full double-precision baseline (black), offering an order-of-magnitude improvement over standard SPFP (red).

The Master (SPFP) code exhibited a drift of +2.22 × 10^–4^ *k*_B_ *T*/ns/DOF. This positive drift, often referred to as “heating,” is typically associated with PME grid jitter.^21,23,35^ The MINT32 implementation reduced this drift to +2.22 × 10^–5^ *k*_B_ *T*/ns/DOF, effectively aligning with the stability of the full double-precision baseline (+2.09 × 10^–5^ *k*_B_ *T*/ns/DOF). This represents a **10**× **reduction in energy drift**. These findings confirm that MINT32’s fixed-point grid offers a significantly more stable foundation for PME charge mapping, effectively mitigating the “grid noise” that affects FP32 simulations.

#### 4.2.2 DHFR Benchmark: Protein Stability

We evaluated four code paths: Master (DPFP), Master (SPFP), MINT32 (Double Bond), and MINT32 (Float Bond). Figure 3 depicts the total energy evolution over the simulation trajectory.

**Figure 3:**
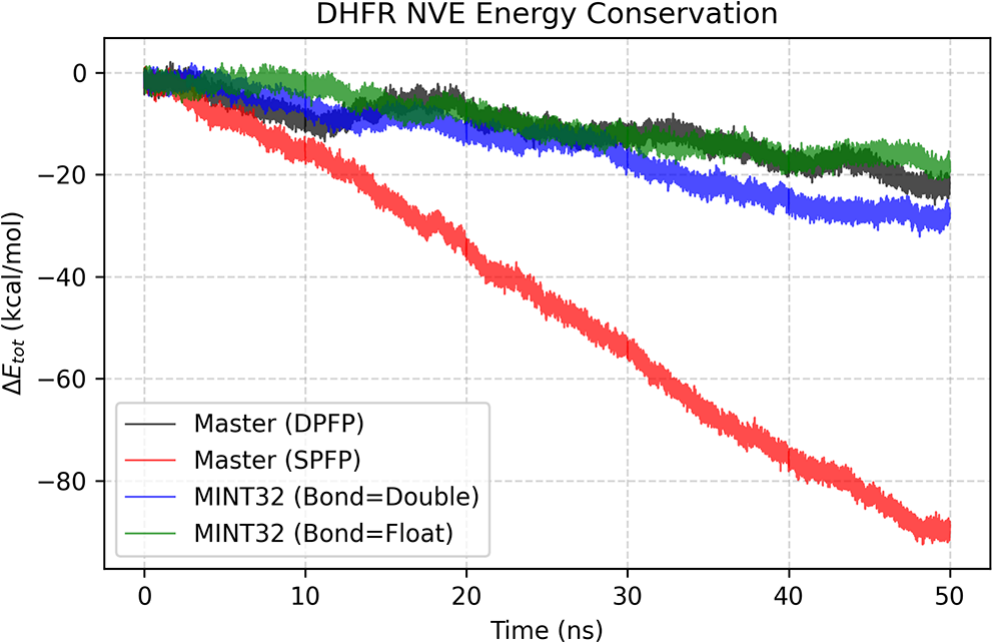
Total energy conservation for the DHFR system (NVE). The MINT32 implementation (blue/green) significantly reduces the energy drift compared with the standard SPFP Master code (red), approaching the stability of the full double-precision baseline (black).

The standard production Master (SPFP) simulation (red line) demonstrated a significant energy drift of −4.64 × 10^–5^ *k*_B_ *T*/ns/DOF. This “cooling” drift is characteristic of FP32 simulations, where the loss of precision at the box edges effectively dampens high-frequency motions. In contrast, the MINT32 implementation with double-precision bonded terms (blue line) reduced this drift to −1.46 × 10^–5^ *k*_B_ *T*/ns/DOF, representing an approximate 3-fold improvement in stability.

Notably, the **MINT32 + Float Bond** variant (green line) achieved a drift of only −9.25 × 10^–6^ *k*_B_ *T*/ns/DOF. This constitutes a **5-fold improvement** over the standard SPFP code and brings the stability within close proximity to the fully double-precision baseline (−8.74 × 10^–6^ *k*_B_ *T*/ns/DOF). This counterintuitive result indicates that once coordinate precision is established by the MINT32 grid, the residual energy drift is dominated by sources other than bonded-force evaluation precision, most likely nonbonded and PME noise. Improving bonded-force precision from FP32 to FP64 therefore provides diminishing returns, while the observation that MINT32 with single-precision bonded terms nearly matches the stability of a full double-precision run strongly supports our hypothesis: coordinate precision, not force evaluation precision, is the predominant source of error.

#### 4.2.3 Factor IX: Larger Protein Benchmark

To test whether the observations from DHFR generalize to larger, compositionally distinct systems, we repeated the four-mode NVE comparison for coagulation Factor IX (90,906 atoms; Figure 4).

**Figure 4:**
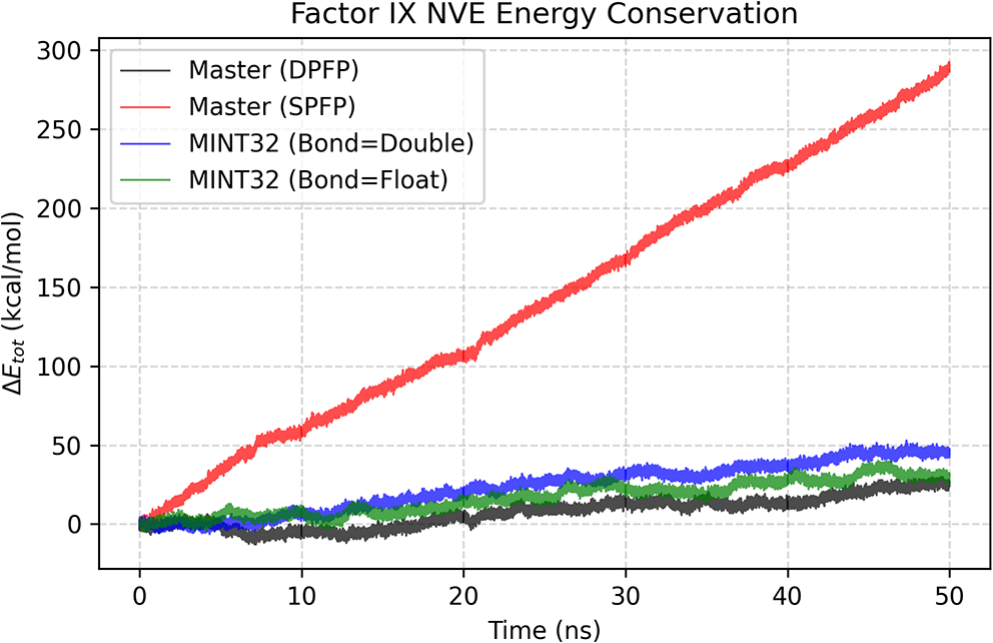
Total energy conservation for the Factor IX system (NVE = 50 ns). The pattern mirrors DHFR: MINT32 variants (blue/green) reduce drift by 5–8× relative to SPFP (red), and the Float Bond variant (green) approaches the double-precision baseline (black).

The Factor IX results closely mirror the DHFR pattern. The Master (SPFP) drift of +3.48 × 10^–5^ *k*_B_ *T*/ns/DOF is reduced to +6.43 × 10^–6^ by MINT32 (Double Bond) and to +4.24 × 10^–6^ by MINT32 (Float Bond), representing approximately **5**× **and 8**× **improvements** over SPFP, respectively. The DPFP baseline drift of +3.78 × 10^–6^ *k*_B_ *T*/ns/DOF confirms that the MINT32 Float Bond variant closely approaches full double-precision stability. Critically, the float-bond advantage observed in DHFR is **reproduced**: the Float Bond drift is 34% lower than the Double Bond drift, consistent with the DHFR finding that once coordinate precision is established, additional precision in bonded-force evaluation provides diminishing returns. The observation that this pattern holds across two protein systems differing in size (∼23K vs ∼91K atoms), composition (DHFR vs a coagulation factor with γ-carboxyglutamic acid modifications), and box geometry (62^3^ vs 142 × 83 × 79 Å^3^) strengthens the generality of the float-bond finding.

### 4.3 PME Grid Alignment Analysis

To provide direct evidence that PME charge-assignment noise is a primary driver of the drift reduction observed in the water system, we analyzed the particle-to-mesh alignment error under both coordinate representations. In PME, a particle at position *x* in a periodic box of length *L* is mapped to a fractional grid coordinate *g* = *xK*/*L*, where *K* is the number of grid points. Charge is distributed to neighboring grid points using fourth-order B-spline interpolation weights that depend on the fractional part of *g*. The coordinate representation error *δx* propagates to a grid-position error δ*g* = δ*x* ·*K*/*L*, and thence to a B-spline weight error bounded by δ*W* ≤ |*M*_4_^′^|_max_ · δ*g* = (2/3)­δ*g*, where 2/3 is the maximum derivative of the fourth-order cardinal B-spline.

For FP32 coordinates in a centered box [−*L*/2, + *L*/2], the worst-case rounding error occurs at the box edge. For all three benchmark systems, the worst-case coordinate falls in the same FP32 exponent range, giving δ*x*_FP32_ = 2^–20^ ≈ 9.5 × 10^–7^ Å. For MINT32, the error is uniform: δ*x*_MINT32_ = *L*/2^33^. The resulting bounds are summarized in Table 4.

**Table 4:** PME Grid Alignment Error Bounds for the FP32 and MINT32 Coordinate Representations^a^

system	δ*g*_FP32_	δ*g*_MINT32_	δ*W*_FP32_	δ*W*_MINT32_	ratio
water (*L* = 49 Å, *K* = 48)	9.3 × 10^–7^	5.6 × 10^–9^	6.2 × 10^–7^	3.7 × 10^–9^	167×
DHFR (*L* = 62 Å, *K* = 64)	9.8 × 10^–7^	7.4 × 10^–9^	6.6 × 10^–7^	5.0 × 10^–9^	132×
factor IX (*L*_*x*_ = 142 Å, *K*_*x*_ = 144)	3.9 × 10^–6^	1.7 × 10^–8^	2.6 × 10^–6^	1.1 × 10^–8^	231×

a δ*g* is the worst-case fractional grid-position error; δW is the corresponding bound on per-particle B-spline weight error $\left(\delta W\leq \frac{2}{3}\delta g\right)$ . For orthorhombic Factor IX, the reported values correspond to the worst axis (*L*_*x*_ = 142 Å, *K*_*x*_ = 144).

MINT32 reduces the worst-case PME grid alignment error by a factor of 132–231× relative to FP32. Under FP32, each particle’s B-spline weights carry representation noise of order 10^–7^ to 10^–6^; when accumulated over thousands of charged particles and propagated through the reciprocal-space energy calculation, this noise produces the nondeterministic “grid jitter” responsible for the elevated drift in the SPFP water benchmark. Under MINT32, the per-particle weight error drops to the order of 10^–9^ to 10^–8^, rendering the charge assignment effectively deterministic at the level of single-precision arithmetic. This is consistent with the observed 10× drift reduction, where the MINT32 drift matches the DPFP baseline, confirming that coordinate-derived PME noise was the dominant source of instability above the double-precision floor.

### 4.4 Constraint Quality

All MINT32 benchmarks were run with a SHAKE tolerance of 10^–7^ Å, 2 orders of magnitude tighter than the default FP32 tolerance of 10^–5^ Å. Rigid water geometry was enforced using SETTLE. As described in the Section 3, both SHAKE and SETTLE operate on MINT32 integer coordinates with FP64 internal arithmetic for all geometry and correction computations; correction displacements are rounded back to INT32 via rint. No constraint convergence failures or SHAKE iteration-count anomalies were observed in any of the production-length benchmarks (100 ns for water; 50 ns for DHFR and Factor IX). The successful completion of these trajectories at the tightened tolerance provides indirect evidence that the MINT32 coordinate grid resolves bond-length differences at the subångström level without the catastrophic cancellation that limits FP32 SHAKE to coarser tolerances. For comparison, OpenMM’s single-precision SHAKE implementation is typically limited to a tolerance of approximately 10^–5^ Å due to FP32 coordinate noise;^11^ the ability to sustain 10^–7^ Å in the MINT32 prototype reflects the improved coordinate precision rather than algorithmic changes to the constraint solver itself. Detailed per-step constraint residuals and iteration statistics are not logged by the current prototype; extracting these diagnostics would require code instrumentation and will be left for future work.

### 4.5 Performance and Overhead

The production AMBER nonbonded kernel processes optimized 16 × 16 atom tiles within a 32-thread warp (8 iterations per tile pair), enabling efficient register reuse and reduced memory traffic. The MINT32 prototype instead uses the older 32 × 32 atom tile layout. This tile-geometry difference, rather than the MINT32 coordinate arithmetic itself, accounts for the speed gap, relative to production.

Measured against the optimized 16 × 16 production code, the MINT32 prototype reaches **84% of production speed** for the water system (415 ns/day vs 495 ns/day). For DHFR and Factor IX, it achieves approximately **87–89% of production speed** (250–255 ns/day vs 288 ns/day for DHFR; 106–108 ns/day vs 121.5 ns/day for Factor IX), confirming that the ratio is stable across a 4-fold increase in system size. These ratios reflect the 32 × 32 vs 16 × 16 tile-geometry difference, not MINT32 arithmetic: integer subtraction is generally as fast as or faster than floating-point subtraction on modern GPUs.

To directly test this attribution, we performed short reference benchmarks using the baseline SPFP engine compiled with the same 32 × 32 warp tiling employed by the MINT32 prototype (these modified configurations are internal development builds and are not available in the public AMBER release). The 32 × 32 SPFP baseline achieved 436.6, 248.6, and 108.2 ns/day for Water, DHFR, and Factor IX, respectively, reaching 86–89% of the optimized 16 × 16 production code. Compared against this tile-matched baseline, the MINT32 prototype incurs negligible additional overhead (0–5%), confirming that the observed 11–16% performance gap relative to production can be attributed to the tile-geometry and warp-synchronous primitive differences rather than to MINT32 integer arithmetic or coordinate conversion costs.

The fact that we achieve double-precision-like accuracy with effectively negligible arithmetic overhead relative to a tile-matched baseline demonstrates the conceptual feasibility of MINT32.

## 5 Discussion

### 5.1 Conceptual Implications for Coordinate Representation Design

The results presented in this study offer high-level methodological insights into the design of coordinate representations for molecular dynamics. The fundamental finding is that the standard IEEE-754 single-precision (FP32) grid is insufficient for modern MD simulations, not due to arithmetic precision per se but due to spatial quantization limits. By mapping the simulation box to a fixed-point integer grid (MINT32), we demonstrated that a uniform spatial resolution of approximately 0.01 fm effectively suppresses the “noise floor” below the level of other integration errors. This suggests that future MD engines should prioritize coordinate representations that guarantee uniform resolution and translational invariance, properties inherent to fixed-point arithmetic but lacking in floating-point formats. Furthermore, the “self-wrapping” behavior of integer arithmetic simplifies minimum-image calculations, indicating that data types can be codesigned with boundary condition logic to eliminate branch divergence in parallel hardware.

### 5.2 Prototype Nature of the AMBER Implementation

The MINT32 implementation described herein is a standalone proof-of-concept prototype. We utilized the AMBER software package solely as a testbed to enable controlled experimentation on numerical precision within a mature MD environment. This implementation is intentionally minimal; the prototype was constructed to isolate the numerical effects of the coordinate representation without the confounding variables of complex production optimizations. Consequently, it should not be interpreted as a proposed feature or an upgrade path for the existing AMBER engine. Production adoption of MINT32-style coordinate representations in any MD engine would require refactoring of the coordinate storage, PME charge mapping, constraint-solver, and trajectory-output pathways—engineering work that is beyond the scope of the present study but feasible for a purpose-built next-generation codebase.

### 5.3 Interpretation of Numerical Results

The numerical results obtained from the 4096 Water, DHFR, and Factor IX benchmarks demonstrate a consistent pattern across all tested systems: MINT32 reduces energy drift by 5–10× relative to standard SPFP, bringing stability to within a factor of 1–2 of the full double-precision baseline. This confirms that for systems spanning 49–142 Å box dimensions and ∼12K to ∼91K atoms, the coordinate noise of FP32 is indeed a dominant source of instability.

The counterintuitive float-bond observation, that MINT32 with single-precision bonded forces achieves *lower* drift than MINT32 with double-precision bonded forces, was observed across both DHFR and Factor IX, systems that differ in size by a factor of 4 and in protein composition. This reproducibility strengthens the interpretation that once coordinate precision is established by the MINT32 grid, the remaining error budget is dominated by nonbonded and PME noise rather than bonded-force evaluation precision. Improving the bonded-force arithmetic from FP32 to FP64 therefore provides no measurable benefit, as the dominant noise sources are unchanged.

The ability to tighten SHAKE tolerances to 1 × 10^–7^ Å in production-length runs further indicates that fixed-point grids can resolve the microscopic inconsistencies that lead to constraint failures in large networks of rigid bonds. While we expect the physics of quantization noise to generalize, the specific drift reduction factors may vary for systems with different force field parameters, integrators, or box sizes.

### 5.4 Energy Drift as a Diagnostic

It is important to note that NVE energy drift at the levels reported here is a diagnostic for numerical quality, not the sole arbiter of simulation usefulness.^36^ Production simulations are almost always conducted in NVT or NPT ensembles, where thermostats absorb the excess energy introduced by numerical noise. The practical consequences of coordinate quantization, therefore, extend beyond drift itself to include constraint convergence, PME charge-assignment determinism, and long-time scale reproducibility. The energy drift metric is valuable precisely because it is a controlled, thermostat-free measure that isolates numerical noise from physical dynamics, enabling the kind of controlled comparison performed in this study.^19^

### 5.5 Complexity Considerations

The MINT32 representation introduces a dual-state architecture (integer coordinates as the authoritative state and floating-point arrays as a derived cache) that adds programmatic complexity relative to a pure floating-point engine. In particular, trajectory output and analysis tools that expect unwrapped Cartesian coordinates require auxiliary bookkeeping, such as wrap-count tracking for quantities such as diffusion coefficients. Similar bookkeeping, however, is already standard practice in production MD engines: AMBER, GROMACS, and OpenMM all maintain separate wrapped and unwrapped coordinate representations, image flags, or box-translation vectors for trajectory postprocessing. The additional complexity of MINT32 is therefore comparable in kind, if not in detail, to the infrastructure already present in mature codebases. Figure 1 and Table 2 make these representation choices explicit across the time step.

### 5.6 Limitations and Non-Goals of This Study

This study was designed with specific constraints to ensure the clarity of the conceptual findings. First, the present implementation uses the older 32 × 32 atom tile layout rather than the optimized 16 × 16 tiles employed by the production AMBER nonbonded kernel, and this architectural difference accounts for the observed 11–16% speed gap relative to production. Tile-matched reference benchmarks confirm that MINT32 integer arithmetic itself contributes negligible additional cost (0–5%; see Section 4.5), so a production implementation adopting MINT32 coordinates within the existing optimized 16 × 16 tile framework would be expected to incur only minimal performance penalty. Second, the prototype implementation supports orthorhombic boxes only because this simplifies both minimum-image logic and coordinate update paths. At the same time, the MINT32 framework extends naturally to nonorthorhombic cells through fractional coordinates, and our worked truncated-octahedron analysis in Section 2.1.3 shows that displacement reconstruction errors remain comparable to FP32 coordinate spacing; a full triclinic implementation, however, remains future work. Third, although NPT behavior is discussed theoretically, our numerical validation was restricted to NVE ensembles so that energy drift could be measured without thermostat or barostat masking. Finally, the benchmark set spans only three systems, from approximately ∼12K to ∼91K atoms, and does not address the behavior of MINT32 in micrometer-scale systems where INT32 range limits could become relevant.

### 5.7 Implications for Next-Generation MD Packages

The primary contribution of this work is to provide empirical evidence and design templates for the architecture of next-generation MD software. As researchers build new engines from scratch, potentially for new hardware targets, the choice of coordinate representation is foundational rather than incidental. The present results suggest that a uniform spatial resolution of approximately 0.01 fm is sufficient to suppress coordinate rounding noise below the level of integrator error for standard biomolecular force fields. For typical simulation boxes below about 200 Å, this resolution can be achieved with a 32-bit integer grid that also handles periodic wrapping naturally through overflow; larger systems may require 64-bit integers or shifted-origin schemes to preserve the same spatial density. More broadly, the data argue that coordinate representation should rank above force-accumulation precision as a design choice because high-precision accumulation cannot recover accuracy already lost in low-precision coordinates. Designers may therefore wish to evaluate MINT32-style grids alongside other alternatives, such as Morton-coded or shifted-origin floating-point schemes, as part of a broader codesign problem involving bandwidth, precision, and dynamic range. We offer this study not as code to be adopted directly but as a methodological guide for those decisions in future software ecosystems.

## 6 Conclusions

In this study, we investigated the numerical limits of single-precision floating-point coordinates in molecular dynamics by developing and testing MINT32, a fixed-point integer prototype. Our analysis and benchmarks on representative stress-test systems reveal that coordinate quantization error establishes a fundamental “noise floor” that compromises stability regardless of force accumulation precision.

Our prototypical implementation demonstrates that a specialized 32-bit integer grid can achieve a “Goldilocks” balance of precision and performance. Key design insights for future MD engines include:

1 **Precision Requirements:** A uniform spatial resolution of approximately 0.01 fm appears sufficient to suppress coordinate noise below the level of integrator error for typical biomolecular systems.
2 **Stability Gains:** Reduced noise in particle positions leads to significant improvements in energy conservation (5–10× reduction in drift across three test systems) and enables tighter constraint tolerances (10^–7^ Å in the reported production runs) without computational penalty. The counterintuitive finding that single-precision bonded forces suffice when coordinates are precise was reproduced across two independent protein systems.
3 **Algorithmic Efficiency:** Co-designing data types with boundary conditions (e.g., using integer overflow for wrapping) can eliminate complex branching logic in parallel kernels.

We emphasize that this work is a conceptual study using AMBER as a testbed. The MINT32 implementation is a minimal research prototype; production-quality integration would require substantial additional engineering across coordinate, PME, and constraint pathways. These findings serve as a methodological guide for the community, suggesting that next-generation MD software architectures should consider fixed-point or hybrid coordinate schemes as a robust alternative to standard floating-point representations.

## Data Availability

The MINT32 implementation detailed in this study is a research prototype incorporated into a development branch of the AMBER software package and is not presently available in the public release. All data, input files, parameter files, and processed outputs required to reproduce the reported results are publicly available in a machine-readable format at https://github.com/taisung/MINT32_Data. The proof-of-concept implementation of the method is available solely for evaluation purposes as a minimal git-style patch against the AMBER master branch snapshot of 11–05–2025, which can be found in the same repository. This patch is provided for reproducibility and evaluation rather than production use.
